# A Combined Filtering Method for ZigBee Indoor Distance Measurement

**DOI:** 10.3390/s24103164

**Published:** 2024-05-16

**Authors:** Zhe Wei, Zhanpeng Zhou

**Affiliations:** School of Computer Science, Civil Aviation Flight University of China, Guanghan 618307, China; findz@cafuc.edu.cn

**Keywords:** RSSI, Zigbee, combined filtering, indoor distance measurement, indoor positioning

## Abstract

Indoor distance measurement technology utilizing Zigbee’s Received Signal Strength Indication (RSSI) offers cost-effective and energy-efficient advantages, making it widely adopted for indoor distance measurement applications. However, challenges such as multipath effects, signal attenuation, and signal blockage often degrade the accuracy of distance measurements. Addressing these issues, this study proposes a combined filtering approach integrating Kalman filtering, Dixon’s Q-test, Gaussian filtering, and mean filtering. Initially, the method evaluates Zigbee’s transmission power, channel, and other parameters, analyzing their impact on RSSI values. Subsequently, it fits a signal propagation loss model based on actual measured data to understand the filtering algorithm’s effect on distance measurement error. Experimental results demonstrate that the proposed method effectively improves the conversion relationship between RSSI and distance. The average distance measurement error, approximately 0.46 m, substantially outperforms errors derived from raw RSSI data. Consequently, this method offers enhanced distance measurement accuracy, making it particularly suitable for indoor positioning applications.

## 1. Introduction

In recent years, with the rapid development of Wireless Sensor Networks (WSNs) [1,2], indoor positioning and distance measurement have become one of the hot topics in the research field. Indoor positioning [3,4,5] has a wide range of application prospects in indoor navigation, object tracking, environmental monitoring, and other fields. However, due to the complexity and diversity of the indoor environment, indoor distance measurement faces a series of challenges, such as multipath effects, signal attenuation, signal blocking, etc. Therefore, how to achieve high-precision, real-time indoor positioning has become a hot spot and challenge in current research.

In indoor distance measurement, the distance measurement method based on ZigBee [6,7,8] has received widespread attention due to its advantages of low power consumption, low cost, and easy deployment. However, the existing ZigBee indoor distance measurement method has problems such as low distance measurement accuracy, large positioning error, poor robustness, etc., which limits its promotion and application in practical applications. Therefore, it is necessary to conduct in-depth research on the existing indoor distance measurement method based on ZigBee to improve the accuracy and reliability of indoor distance measurement.

This study aims to rectify the shortcomings of current indoor ranging methodologies relying on ZigBee by introducing a combined filtering approach, alongside conducting pertinent theoretical analysis and experimental validation. Initially, it investigates the impact of Zigbee’s indoor transmission power and channel on the RSSI value. Subsequently, it applies linear fitting to the RSSI data to quantify errors in unfiltered conditions. Following this, an indoor ranging scheme employing a combined filtering algorithm is proposed to mitigate these errors. Finally, through a blend of experimental simulations and real-world scenario testing, the efficacy and performance advantages of this method are validated. Through the research conducted in this study, it is anticipated to offer novel insights and methodologies for the advancement of indoor distance measurement technology, thereby fostering the adoption and application of indoor positioning technology based on ZigBee in practical scenarios.

The remainder of this research is structured as follows: Section 2 delves into the related work. In Section 3, the proposed KDGM combined filtering algorithm is elaborated upon. Section 4 provides a detailed description of the experimental procedure and an analysis of the results. Section 5 outlines future directions, while Section 6 concludes this work.

## 2. Related Work

Advancements in positioning technologies have surged in recent years, with a primary focus on enhancing accuracy and precision across diverse applications. Within this dynamic landscape, numerous studies have emerged, each contributing unique methodologies and insights to the field. For instance, ref. [9] introduces a collaborative positioning method tailored primarily for underground mining applications. While its scope may appear specific, its methodology offers insights applicable to refining algorithms and bolstering precision in the design of indoor positioning systems. In a similar vein, ref. [10] directly addresses indoor positioning, employing a diverse array of algorithms integrating Time of Arrival (ToA) and Inertial Measurement Units (IMUs) to achieve high-precision indoor positioning. This study serves as an important resource for guiding the selection and optimization of sensor fusion algorithms in indoor positioning. Moreover, ref. [11], while not directly centered on indoor positioning, introduces a rapid advancement method that provides techniques for enhancing positioning speed and optimizing algorithm performance—crucial elements for the development of indoor positioning systems aiming to deliver efficient and real-time services. Furthermore, ref. [12] delves into an enhanced vehicle-tracking scheme, taking into account environmental factors in driving motions. This scheme effectively tracks moving vehicles on the road using at most two successive base stations (BSs), enabling the associated data analysis platform to promptly furnish up-to-date information about the position of each vehicle.

While existing studies have made significant strides in improving positioning accuracy and precision, our study takes a closer look at indoor distance measurement. In the following discussion, we delve into the related models and methods related to our proposed method.

### 2.1. Path Loss Model

The principle of RSSI distance measurement is based on a certain relationship between the received signal strength and the distance. When the signal propagates from the transmitter to the receiver, it will be attenuated due to various factors, including free-space transmission loss, multipath effects, obstacles, signal attenuation, etc. RSSI distance measurement uses this attenuation relationship to estimate the distance between communication devices by measuring the received signal strength. The common RSSI signal propagation models currently include the Free-Space Path Loss (FSPL) [13], Log-Distance Path Loss Model [14], and Shadowing Model [15]. In indoor environments, the Log-Distance Path Loss Model is more commonly used and applicable. This is because the indoor environment has a more complex structure, with many obstacles, walls, furniture, etc., which cause multipath propagation and reflection of the signal, leading to random fluctuations in signal strength. The formula for the Log-Distance Path Loss Model is as follows:

(1) $$PL\left(d\right)=PL_{0}+10\cdot n\cdot \mathrm{lg}\left(\frac{d}{d_{0}}\right)+X_{\sigma },$$

where $PL\left(d\right)$ is the RSSI between the transmitter and receiver when the distance is d, $PL_{0}$ is the RSSI at the reference distance $d_{0}$, n is the path loss factor, which is affected by the actual site environment, and $X_{\sigma }$ is a Gaussian random variable with a mean of 0 and a standard deviation of σ (usually taken as 4–10 dB) [16]. In actual use, $d_{0}$ is often taken as 1 m, so the model can be simplified as follows:

(2) RSSI(d)=A−10⋅n⋅lgd,

where A represents the received signal strength at a distance of 1 m from the position node, and RSSI(d) is the signal strength value at a distance of d m. Based on this formula, the distance equation can be derived as follows:

(3) $$d=10^{\frac{A-RSSI(d)}{10\cdot n}}.$$

### 2.2. Mean Filtering

Mean filtering [17] is a simple and low-cost computational technique, which involves summing and then averaging the data set collected at a specific location. This method is very suitable for implementation on the resource-limited CC2530 microcontroller, as its 256 KB of storage space and 32 MHz clock frequency are sufficient to handle the computational requirements of average filtering. The formula is as follows:

(4) $$RSSI=\frac{1}{n}\sum_{i=1}^{n}\;RSSI_{i},$$

where RSSI represents the strength of the received signal, *n* is the number of samples of signal strength, and $RSSI_{i}$ represents the signal strength value of the i-th sample. This model can smooth the signal well for certain Gaussian white noise and retain the general trend of the signal. However, when the value of *n* is large enough, there is a certain lag in the filtering of RSSI, which slows down the overall response speed of the system.

### 2.3. Kalman Filtering

Kalman filtering [18] is a recursive method used to estimate the state of dynamic systems. It updates the state estimate and state covariance matrix by integrating the system model with observed data to produce an optimal estimate. This capability is essential for processing signals in environments that are dynamic and constantly changing. Implementing the Kalman filter on the CC2530 microcontroller is completely viable. The CC2530 offers sufficient computational power and memory (8 KB RAM) to manage the basic matrix operations essential for Kalman filtering. Despite its limited resources, by strategically simplifying and optimizing the algorithm—for instance, by utilizing streamlined data structures and processes—the filter can be executed both efficiently and stably. Kalman filtering operates through two principal stages: prediction and update.

In the prediction stage, the dynamic model is used to predict the system’s state through the following equation:

1. State prediction:
  (5) $$x_{k}=A_{k}\cdot x_{k-1}+B_{k}\cdot u_{k},$$
  where $x_{k}$ represents the estimated state at time k, $A_{k}$ is the state transition matrix, $B_{k}$ is the control input matrix, and $u_{k}$ is the control input.
2. Error covariance prediction:
  (6) $$P_{k}=A_{k}\cdot P_{k-1}\cdot A_{k}^{T}+Q_{k}.$$
  This equation predicts the error covariance, where $P_{k}$ is the current error covariance matrix, and $Q_{k}$ represents the process noise covariance matrix.

In the update stage, the prediction is fused with the observed data by calculating the Kalman gain and then correcting the state and error covariance by using the following equations.

3. Calculate the Kalman gain:
  (7) $$K_{k}=P_{k}\cdot H_{k}^{T}\cdot (H_{k}\cdot P_{k}\cdot H_{k}^{T}+R_{k}{)}^{-1},$$
  where $H_{k}$ is the observation matrix, and $R_{k}$ is the observation noise covariance matrix.
4. State correction:
  (8) $$x_{k}=x_{k}+K_{k}\cdot (z_{k}-H_{k}\cdot x_{k}),$$
  where $z_{k}$ represents the observed value.
5. Error covariance correction:
  (9) $$P_{k}=\left(I-K_{k}\cdot H_{k}\right)\cdot P_{k},$$
  where I is the identity matrix.

These Formulas (5)–(9) are central to Kalman filtering, enabling recursive estimation by integrating predictions and observations to improve the accuracy of state estimates and error correction.

### 2.4. Gaussian Filtering

Gaussian filtering [19] effectively suppresses noise by using the Gaussian function as a weighting function and smoothing the signal using its probability density function. Implementing Gaussian filtering on the CC2530 microcontroller is entirely feasible, although it requires some complex mathematical operations. Given that our research does not require high real-time data processing, the processing power of the CC2530 is fully capable of meeting the needs of Gaussian filtering. Its excellent noise suppression characteristics make Gaussian filtering a preferred option, suitable for use in resource-limited environments. Its probability density function formula is as follows:

(10) $$\phi (x)=\frac{1}{\sqrt{2\pi }\sigma }e^{-\frac{(x-\mu {)}^{2}}{2\sigma^{2}}},$$

among which

(11) $$\mu =\frac{1}{n}\sum_{i=1}^{n}\;x_{i},$$

(12) $$\begin{array}{c} \sigma =\sqrt{\frac{1}{n-1}\sum_{i=1}^{n}\;{\left(x_{i}-\mu \right)}^{2}} \end{array}.$$

In this study, the probability interval of 0.68 ≤ *ϕ*(*x*) ≤ 1 was chosen for analyzing RSSI signal strength values. Selecting this interval implies focusing on RSSI values within ±1*σ* of the mean. This range encompasses the most representative data, as these values are tightly clustered around the overall average. Gaussian filtering effectively captures the consistent and prevalent signal strength characteristics in the environment, while ignoring outliers that might be caused by drastic environmental fluctuations, device inaccuracies, or other random occurrences.

### 2.5. Dixon’s Q-Test

Dixon’s Q-Test [20] method is a statistical method used to identify and remove outliers from a data set, which plays a key role in improving distance measurement accuracy. Especially when using RSSI data for distance estimation, the presence of outliers can severely affect the accuracy of the results. In the context of using the CC2530 microcontroller, choosing Dixon’s Q-test is particularly appropriate because this test method does not require complex calculations and is suitable for execution on resource-limited devices. The steps for applying Dixon’s Q-Test to filter RSSI values are as follows:

Step 1: Collect n RSSI data points and arrange them in ascending order as $RSSI_{1}\leq RSSI_{2}\leq RSSI_{3}\leq \ldots \leq RSSI_{n}$; then, determine the significance level α=0.05 [21].

Step 2: Depending on the sample size (in this case, 30), use the Dixon’s Q-Test formulae to check for high- and low-end outliers. For sample sizes between 14 and 30, the formulas are

(13) $$D_{n}=\frac{x_{\left(n\right)}-x_{\left(n-1\right)}}{x_{\left(n\right)}-x_{\left(3\right)}}\;{D_{n}}^{\prime }=\frac{x_{\left(3\right)}-x_{\left(1\right)}}{x_{\left(n-2\right)}-x_{\left(1\right)}},$$

where $D_{n}$ is the statistic for testing high-end values, and ${D_{n}}^{\prime }$ is the statistic for testing low-end values.

Step 3: In this study, with α=0.05, obtain the corresponding critical value in the Dixon’s Q-Test critical value table D(α,n).

Step 4: Compare the calculated Dixon’s Q-Test statistic with the critical value. If the Dixon’s Q-Test statistic exceeds the critical value, it can be concluded that there are outliers in the data set.

Step 5: After removing the outliers, repeat the previous steps with the remaining RSSI values until no outliers are present.

## 3. KDGM Combined Filtering Algorithm

In practical RSSI measurement, it is found that using a single filtering algorithm still results in a large number of outliers. These outliers may be caused by environmental factors, equipment failures, or other unknown factors, which seriously affect the accuracy and stability of the data. Therefore, when facing these problems, it is necessary to adopt a more comprehensive and effective method to process RSSI data to improve the accuracy and reliability of the measurement. Based on this, this article proposes a KDGM (Kalman–Dixon–Gaussian–Mean) combined filtering algorithm, aiming to optimize the measured RSSI values by combining the advantages of multiple filtering technologies. The design inspiration of this algorithm comes from the characteristics of various filtering algorithms at different data processing stages, such as the dynamic system model of Kalman filtering, the outlier detection capability of Dixon’s Q-Test, the data smoothing effect of Gaussian filtering, and the data fusion capability of mean filtering. The reasons for choosing the KDGM combined filtering algorithm are as follows:

1. Resource constraints:
  Microcontrollers typically have limited computational resources and memory. Specifically, the CC2530 utilizes an 8-bit enhanced 8051 microcontroller core, where computational capacity is restricted. Thus, selecting algorithms with relatively low complexity and moderate computational demands is more practical. Mean filtering and Gaussian filtering are straightforward to implement in resource-limited environments and offer high computational efficiency, making them suitable for microcontroller applications.
2. Real-time requirements:
  Microcontrollers are often used in real-time systems, necessitating algorithms that can respond quickly. Kalman filtering offers an effective method for real-time estimation and correction of system states, making it suitable for real-time tracking and prediction in dynamic systems.
3. Outlier handling:
  Dixon’s Q-test filtering is effective in identifying and handling significant outliers, making it suitable for data potentially affected by occasional anomalies. This capability is crucial for ensuring data accuracy and reliability.
4. Data characteristics:
  Gaussian filtering can smooth data, reducing the impact of noise without overly altering the inherent characteristics of the data. This property makes it suitable for processing signals with Gaussian noise characteristics.

**Figure 1:**
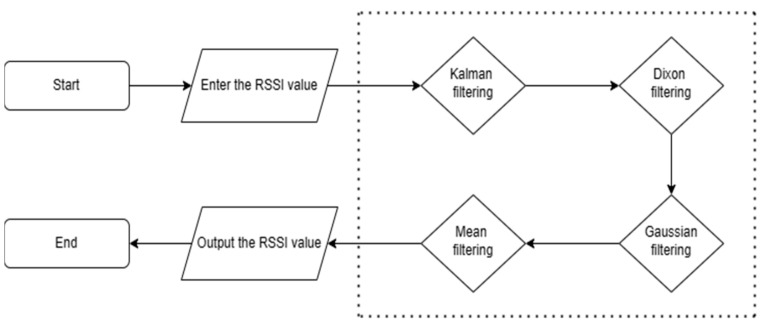
Flowchart of the KDGM combined filtering algorithm.

In addition, the filtering process is described as follows, as shown in Figure 1:

1. First, based on the Kalman filtering algorithm, the initial RSSI value is filtered. The Kalman filtering algorithm is a recursive filtering technology that can effectively eliminate noise and uncertainty by estimating and correcting the state of the system, thereby improving the accuracy and stability of the data.
2. Secondly, for the RSSI value after Kalman filtering, Dixon’s filtering is performed. According to the significance level α=0.05, the calculated Dixon statistic is compared with the critical value until there are no outliers. Dixon’s filtering method is a statistical method used to identify outliers. By calculating the difference between the sample data and the median of the sample, and comparing it with other samples, outliers are detected and removed, thereby reducing data errors and biases.
3. Subsequently, for the RSSI value after Dixon’s filtering, the Gaussian filtering algorithm is used for smoothing, and the RSSI value falling in the probability $0.68\leq F_{RSSI}\leq 1$ interval is selected. Gaussian filtering is a filtering method based on Gaussian distribution. By performing weighted averaging or convolution operations on the data, the original data are smoothed, thereby eliminating noise and sudden interference and improving the stability and reliability of the data.
4. Finally, for the RSSI value after Gaussian filtering, the average is accumulated and taken to obtain the final RSSI value. Through accumulation and averaging operations, the accuracy and stability of the data can be further improved, measurement errors can be reduced, and the strength and distance of the wireless signal can be more accurately reflected.

The KDGM combined filtering algorithm is shown in Algorithm 1.
**Algorithm 1:** KDGM combined filtering**Input:** RawRssi (raw RSSI data), Distance**Output:** avgRSSI (average RSSI after filtering) 1: Apply the Kalman filter to RawRssi and round the result to get kalman_result 2: Set distanceValue to Distance 3: Set DixonsDistance to DistanceValue and DixonsRssi to kalman_result 4: Set the batch size for Dixon’s Q-Test 5: Initialize the final RSSI value list, final_rssi 6: **for** each batch of data in DixonsRssi **do** 7:    Get the current batch of data, batch_data 8:    Apply Dixon’s Q-Test filter to batch_data to get filtered_rssi 9:    **if** filtered_rssi is not empty **then**         Compute the mean of filtered_rssi and add it to final_rssi 10:    **end if** 11: **end for** 12: Set GaussuanDistance to DixonsDistance and GaussuanRssi to final_rssi 13: Apply the Gaussian filter to GaussuanRssi to get gaussian_result 14: Compute the mean of gaussian_result and round it to get avgRSSI 15: **return** avgRSSI

## 4. Experiment and Result Analysis

In this section, we analyze the possible sources of error in indoor distance measurement, conduct experiments on Zigbee’s transmission power, wireless channel, and height from the ground, find suitable parameters, and reduce the impact of parameter settings on RSSI values. Next, we fit the parameters of the indoor path loss model to obtain the A and n values suitable for the current environment, and finally, we verify the filtering performance of the KDGM combined filtering algorithm through experimental testing.

### 4.1. Analysis of Indoor Distance Measurement Error Sources

Using RSSI to achieve indoor distance measurement is a common method at present, but the accuracy of Zigbee’s indoor distance measurement will be affected by the multipath propagation of the signal, signal attenuation, and non-line-of-sight (NLOS) [22,23] propagation caused by obstacles in the propagation process, which will produce the following problems:

Random jumping of RSSI

When the radio signal propagates in the air, it may be subject to RSSI jumps caused by other devices or electromagnetic interference. This interference may be temporary or persistent. Also, when the wireless signal reaches the receiver through multiple paths, it may also cause rapid changes in signal strength. These situations are common problems in urban environments [24].

Inaccurate environmental parameter values of the distance measurement model [25]

The parameters A and n of the distance measurement model play a crucial role in determining the quality of the model. Parameter A represents the reference signal strength at a given distance, typically indicating the signal strength at one meter from the source. Parameter n, known as the path loss exponent, reflects how much the signal attenuates as it propagates over a distance. This parameter can vary depending on environmental factors such as indoor versus outdoor settings, building structures, and obstructions.

In different indoor environments, the values of A and n can vary significantly, as shown in Table 1. Relying solely on traditional experience to set these two values as fixed values can lead to large errors in estimating the distance. This discrepancy occurs because fixed values may not accurately capture the unique characteristics of specific environments, resulting in an estimated distance that does not align with the actual indoor environment distance.

Zigbee device parameter settings

The transmission power of Zigbee [26] and the channel selection in the indoor environment are also particularly important. The size of the transmission power determines the length of the communication distance and the penetration ability, and the channel selection determines the network performance and stability.

Impact of node distance from ground height on RSSI

The RSSI may also be affected by the distance from the ground [27]. If both the transmitter and the receiver are on the ground, they may be affected by ground reflection. Therefore, it is necessary to place the Zigbee nodes [28] at different height intervals on the ground, measure the RSSI, and observe the impact of height on the RSSI.

**Table 1:** A and N correspondence table for different scenarios [29].

Scene	A	N
Park	32.7~36.0	2.7~3.4
Stair	35.0~38.2	1.9~2.5
Grassland	33.2~36.4	3.0~3.9
Beach	37.5~40.8	3.8~4.6
Office	39.0~50.5	1.4~2.5

Therefore, it is necessary to first test the indoor transmission power and channels, etc., to find parameters that fit the current environment.

### 4.2. Indoor Transmission Power Test

The indoor short-distance transmission power test uses the SmartRF-Studio7 [30,31] developed by TI. All Zigbee nodes use a rubber stick antenna with a gain of 3 dBi. The test frequency is 2405 MHz, the channel is the default Channel 11, and the receiver sensitivity is −97 dBm. The experiment was conducted in a computer room laboratory.

The test mode is set to Expert Mode, and the transmission power setting range is −22 dBm to 4.5 dBm. Each time, 30 packets are sent, and each packet is spaced 1000 milliseconds apart.

The receiver settings are the same as above, and the expected number of received packets is set to 30. In Dump Data to File, the save path for each received data point is set.

**Figure 2:**
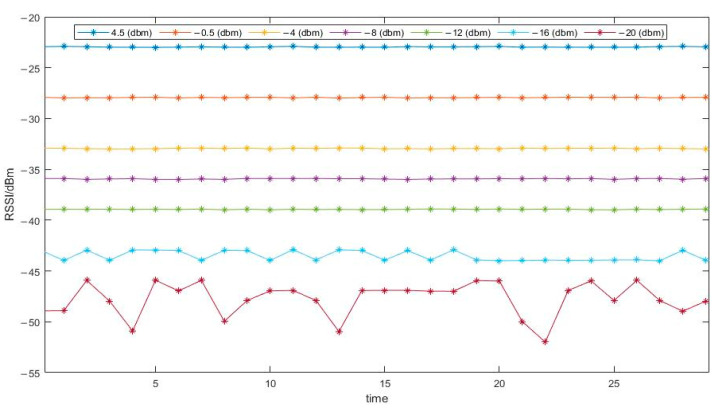
Effect of transmission power on RSSI.

Two Zigbee nodes are fixed at different positions, keeping the test distance constant at 1 m. The transmission frequency is set to 2405 MHz, and the transmission power [32] is set to −20 dBm, −16 dBm, −12 dBm, −8 dBm, −4 dBm, −0.5 dBm, and 4.5 dBm. Experiments are conducted under these seven fixed transmission powers. The experimental results are shown in Figure 2.

As can be seen from the figure, under the same transmission frequency, different transmission powers have a significant impact on the RSSI value. A higher transmission power can not only enhance the reliability of the RSSI value but also increase its stability.

### 4.3. Wireless Channel Test

**Figure 3:**
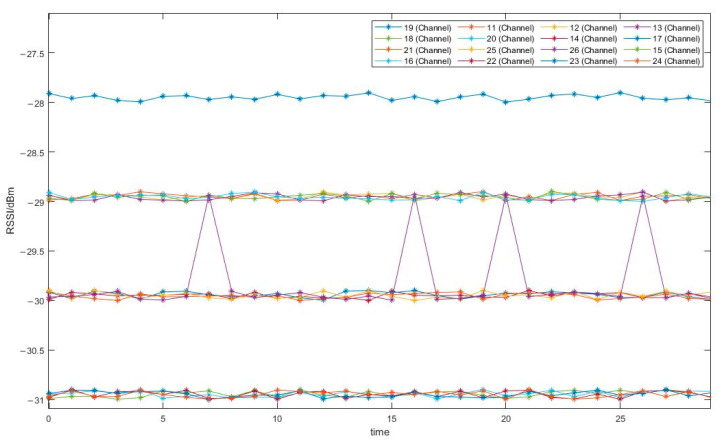
Effect of channel on RSSI.

In the same experimental environment as in Section 4.2, the transmission power is set to 1 dBm, and Zigbee’s channels 11 to 26 in the 2.4 G frequency band are tested. The experimental results are shown in Figure 3.

In the experiment, there are situations where the RSSI values are the same under different channels [33,34]. In order to better observe the data, a random offset of 0.1 is added when generating the line graph for easy observation of the results. As can be seen from the figure, in the indoor environment, due to the influence of multipath propagation and reflection, the choice of different channels will also affect the RSSI. Among them, Channel 19 performs well in this experimental environment.

### 4.4. Zigbee Height from Ground Test

**Figure 4:**
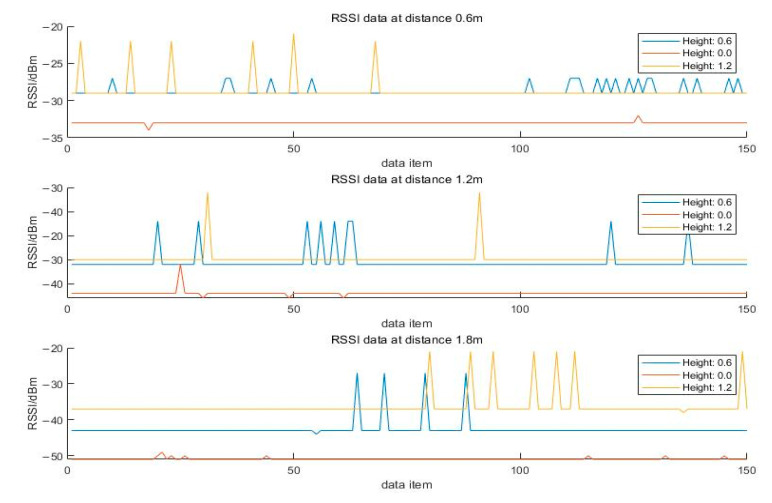
Plot of RSSI measurements at different altitudes.

Zigbee devices are placed at three different heights for the experiment, which are 0 m, 0.6 m, and 1.2 m. The distances between the receiver and the transmitter are set to 0.6 m, 1.2 m, and 1.8 m. At each distance point, 150 data points are collected. Zigbee nodes are all fixed for the experiment using tripods. The data measured at different distances under different heights are shown in Figure 4.

As can be seen from the figure, the height of the node from the ground has a certain impact on the RSSI value. This impact is significant when the distance between the nodes increases. In this experiment, when the node is 1.2 m from the ground, the RSSI value is significantly better than when it is at 0.6 m and 0 m. This proves that increasing the height of the Zigbee node can reduce the reflection of the signal from the ground, making the signal superior to when it is close to the ground.

### 4.5. Path Loss Model Fitting

In the computer room laboratory environment, the router node is placed on a bracket at a height of 0.8 m. The node to be tested is moved at distances from 0.3 m to 3.4 m. At each location, 1500 RSSI data points are collected. The node to be tested sends data frames to the router node. The router node forwards the read RSSI values to the coordinator node. The coordinator then establishes data transmission with the PC through serial communication. In the end, a total of 14 distance points were collected, totaling 21,000 RSSI data points.

**Figure 5:**
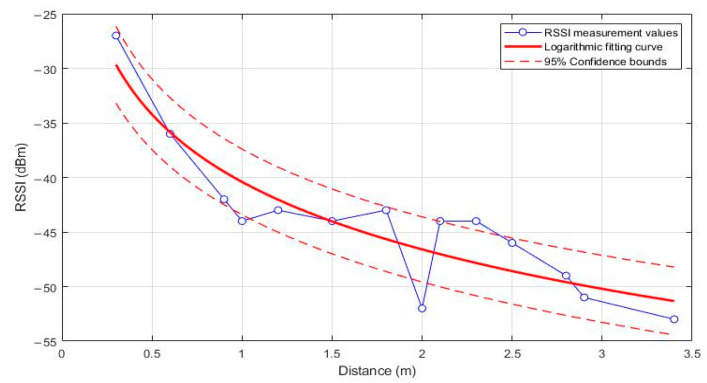
Fitted curve.

As can be seen from Formula (3), the values of the constant A and n will affect the calculation of converting RSSI values into distance values. These two values are mainly determined by the current environment. The traditional method is to treat n as a constant. However, due to the interference of multipath effects in the environment, the value of n changes with the environment. In order to ensure that the values of A and n do not affect the distance measurement accuracy of RSSI, it is necessary to perform logarithmic fitting on the parameter values. The collected 14 sets of data are filtered, and then the least squares method is used for curve fitting, as shown in Figure 5.

As illustrated in Figure 5, the relationship between the RSSI and distance was modeled using a logarithmic fit, with a coefficient of determination of 0.83, indicating a significant functional relationship between RSSI values and distance. The fitted parameters of the model are *A* = −40.41 and *n* = 2.05, reflecting the expected attenuation of signal strength with increasing distance.

Further analysis, supported by the 95% confidence intervals, reveals that the variations in RSSI values are most pronounced within the 1–1.5 m range. The choice of a 95% confidence interval is guided by the standard practice in statistical analysis, which strikes a balance between providing sufficient assurance that the interval estimates the true parameter values while not being overly conservative. This level of confidence is widely accepted in scientific research, as it offers a high degree of certainty without being excessively stringent, thereby providing a reliable basis for decision-making in practical applications.

These confidence intervals further validate the predictive accuracy of the model and demonstrate the high reliability of the measurements over this distance range. In addition, while the overall performance of the model is good, the analysis of the confidence intervals also shows that the variation in RSSI values flattens out beyond 1.5 m. This observation suggests that for subsequent applications of RSSI-based distance measurements involved in the current environment, a maximum effective measurement distance of 1.5 m should be considered to ensure high accuracy and reliability of the measurements.

### 4.6. KDGM Combined Filtering Algorithm Experiment and Result Analysis

**Figure 6:**
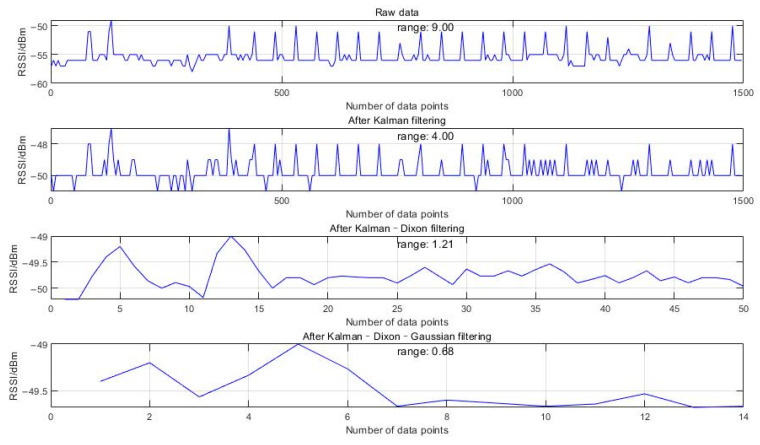
Comparison between the results of filtering algorithms.

The transmitter (coordinator) was placed on a tripod at a height of 1.2 m and connected to a PC; then, the receiver (terminal) was placed at a fixed distance from the transmitter for the experiment. The collected data set was processed separately through Kalman filtering, Kalman–Dixon filtering, and Kalman–Dixon–Gaussian filtering. The filtered RSSI values processed by MATLAB are shown in Figure 6.

The figure illustrates the impact of various filtering techniques on the fluctuations in the Received Signal Strength Indicator (RSSI) data. The raw RSSI data exhibit significant variability, with a fluctuation range of 9.00, potentially caused by environmental interference or equipment variability. By applying Kalman filtering, we observed a notable reduction in the fluctuation range to 4.00, demonstrating the effectiveness of Kalman filters in reducing noise in dynamic systems predicted based on time-series data. Further integration of the Dixon’s Q-test into Kalman–Dixon filtering reduced the range to 1.21. The Dixon’s Q-Test, an effective outlier detection method, helped further eliminate extreme data points, thus optimizing signal smoothness. Finally, incorporating Gaussian filtering into the Kalman–Dixon filter further reduced the fluctuation range to 0.68. Gaussian filtering focuses on local consistency and smoothing out short-term fluctuations by averaging nearby data points with weighted importance, further enhancing overall data stability.

Variance measures the degree of deviation between a random variable and its mean. As shown in Table 2, the deviation of the RSSI values from the mean is minimized after the KDGM combined filtering algorithm, indicating that the filtering effect meets the requirements.

### 4.7. RSSI Distance Measurement Comparison Test

**Figure 7:**
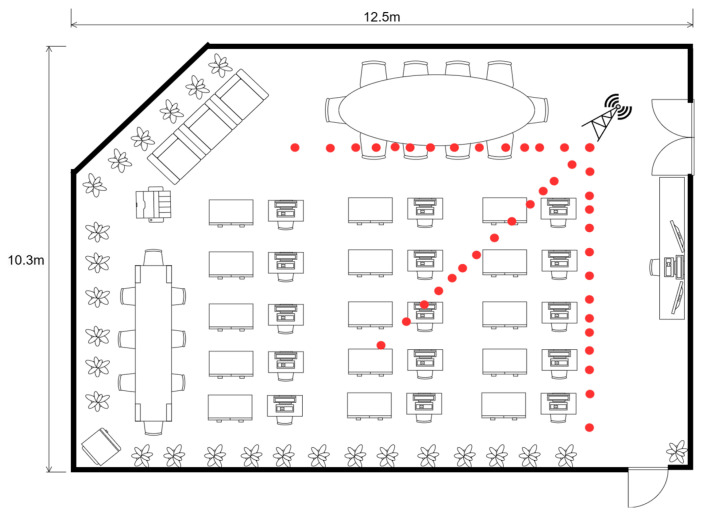
The layout of the testing site.

Initially, the ranging experiments were conducted within a computer laboratory space measuring 12.5 m by 10.3 m. Drawing upon the outcomes from prior investigations concerning Zigbee base height, transmission power, and wireless channel configurations, the experimental setup was optimized by setting the antenna height at 1.2 m, the transmission power to 4.5 dBm, and selecting channel 19 for the wireless communication to mitigate the impact on RSSI values significantly. As depicted in Figure 7, a coordinator node was strategically deployed within the laboratory environment, with terminal nodes precisely positioned at fourteen data collection points to ensure comprehensive coverage.

**Figure 8:**
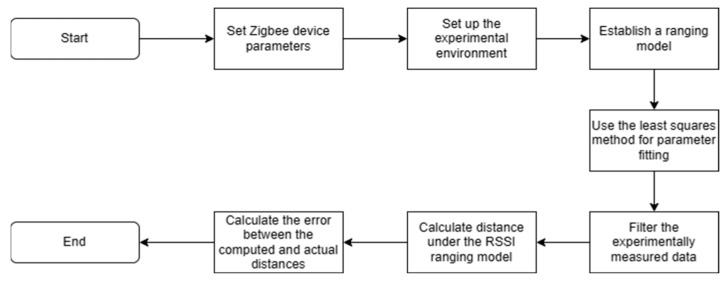
Ranging process.

To circumvent the potential biases introduced by non-omnidirectional antennas, RSSI measurements were gathered from three distinct angles, and subsequently, data from identical distances but differing directions were amalgamated to enhance accuracy. The ranging process is shown in Figure 8.

**Figure 9:**
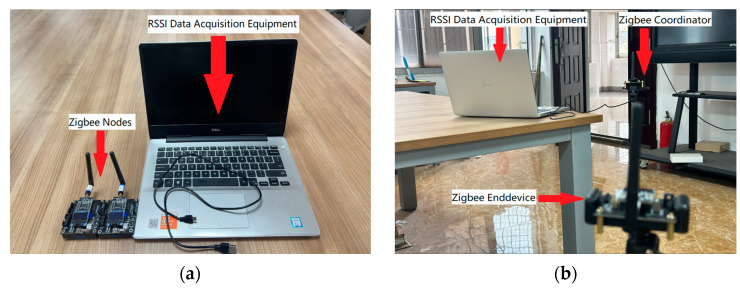
Experimental equipment.

The experiments utilized Zigbee nodes equipped with CC2530 chips from Texas Instruments, known for their reliability and performance in wireless communications. As illustrated in Figure 9, subfigure a shows the devices used for collecting RSSI data and the two Zigbee nodes, while subfigure b illustrates the Zigbee coordinator node and end node during the experiment, the RSSI data collection apparatus was interfaced with the Zigbee coordinator via a serial connection, ensuring robust data transmission. This study employed two Zigbee nodes to facilitate the thorough collection of RSSI data between the designated transmitter and receiver. Table 3 presents a detailed comparison between the raw RSSI data and the distance estimation results following the application of the KDGM combined filtering, highlighting the methodological efficacy and enhancement in measurement accuracy.

Through comparison of the “RSSI Raw Data” and “KDGM-Filtered Data” columns, it is observed that the measured distance errors are generally reduced after KDGM filtering. In particular, at short distances, the filtered data exhibit a more stable and accurate trend, indicating a significant effect of the KDGM filtering algorithm in reducing noise and interference in the ranging system. However, in some cases, especially at longer distances, measurement errors still show some degree of fluctuation. This may be attributed to environmental noise or other external factors that have not been fully eliminated. In summary, the analysis of the data in Table 3 indicates the potential of the KDGM combined filtering algorithm in optimizing ranging system performance, providing important references and guidance for future research and engineering practice.

**Figure 10:**
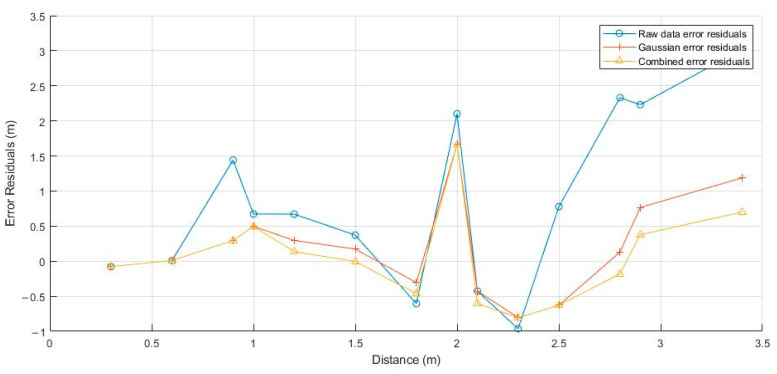
Comparison of ranging errors with and without filtering algorithms.

Figure 10 shows the error residual plot for data without filtering and with filtering algorithms applied.

As can be seen from the figure, the error is the largest when no filtering is applied. After filtering, the error decreases, and the KDGM combined filtering algorithm has the smallest error. This indicates that after filtering, the accuracy of indoor ranging has greatly improved. The average error is shown in Table 4.

This study systematically evaluated three indoor distance measurement error processing methods: unfiltered data, Kalman–Dixon filtering, and KDGM combined filtering algorithm. The results showed that the latter two advanced filtering techniques significantly improved measurement accuracy. The unfiltered data exhibited the highest mean error (1.12 m) and variance (1.50 m^2^), indicating substantial measurement uncertainty and instability. The introduction of Kalman–Dixon filtering reduced the mean error and variance to 0.52 m and 0.46 m^2^, respectively, highlighting its effectiveness in reducing random fluctuations and enhancing data consistency. The KDGM combined filtering algorithm further reduced the mean error and variance to 0.46 m and 0.41 m^2^, with its advanced data processing techniques not only improving measurement precision but also enhancing robustness in dynamic environments. KDGM’s integration of robust statistical methods with real-time filtering algorithms enables it to outperform traditional methods in terms of accuracy and consistency. These findings provide a strong theoretical and empirical basis for the future application of this technology in more complex environments.

## 5. Future Directions

The complex physical structure of indoor environments often leads to signal reflection and multipath effects. While the method proposed in this study can effectively reduce these errors, enhancing the accuracy and reliability of distance measurements, it may not be directly applicable to outdoor settings. In outdoor environments, environmental factors such as weather and extensive physical obstacles may significantly affect signal transmission, thereby impacting distance measurement accuracy. To meet broader application needs, it is advisable to adjust these filtering methods or integrate other technologies to enhance performance and applicability in outdoor environments. Specifically, the introduction of machine learning algorithms such as Support Vector Machine (SVM) and deep learning networks can automatically adjust filtering parameters by learning environmental features, thus adapting to complex outdoor environmental changes. Additionally, combining Global Positioning System (GPS) and Long-Range Wide-Area Network (LoRaWAN) technologies could be an effective solution, providing wider coverage and higher positioning accuracy. Sensor fusion techniques could also be considered, integrating data from Inertial Measurement Units (IMUs), magnetometers, and visual sensors to achieve more precise and stable positioning outputs. The combination of these technologies might not only enhance system robustness but would also maintain efficient performance in diverse outdoor environments.

## 6. Conclusions

This study addresses indoor distance measurement errors by introducing the KDGM combined filtering algorithm. Our experiments comprehensively explore the impact of transmission power, channel selection, signal propagation models, and filtering algorithms on RSSI values. The results emphasize the significance of these factors in achieving accuracy in indoor distance measurement. Through the effective elimination of outliers in the RSSI data, the KDGM algorithm enhances distance measurement accuracy for Zigbee nodes. However, further enhancements are necessary to better align with practical application requirements across diverse environmental conditions. Future research endeavors could also delve into the broader application scope of the KDGM algorithm and optimize its parameters to address a wider range of measurement needs and environmental challenges.

## Figures and Tables

**Table 2:** Variance comparison table.

Filtering Methods	Raw Data	Kalman-Filtered	Kalman–Dixon-Filtered	KDGM-Filtered
Variance	2.40	0.41	0.05	0.04
Range	9.00	4.00	1.21	0.68

**Table 3:** Comparison of distance measurement errors.

	RSSI Raw Data			KDGM-Filtered Data		
Actual Distance (m)	RSSI (dBm)	Measured Distance (m)	Error (m)	RSSI (dBm)	Measured Distance (m)	Error (m)
0.9	−48	2.34	1.44	−42	0.22	0.30
1	−45	1.67	0.67	−44	0.61	0.50
1.2	−46	1.87	0.67	−43	1.20	0.14
1.5	−46	1.87	0.37	−44	1.50	0.00
1.8	−42	1.20	0.60	−43	1.34	0.46
2.1	−53	4.10	2.00	−52	3.67	1.57
2.3	−45	1.67	0.63	−44	1.50	0.80
2.5	−43	1.34	0.96	−44	1.50	0.81

**Table 4:** Comparison of mean error and variance.

Filtering Methods	Not Filtered	Kalman–Dixon-Filtered	KDGM-Filtered
Mean Error (m)	1.12	0.52	0.46
Variance (m^2^)	1.50	0.46	0.41

## Data Availability

The data used to support the findings of this study are available from the corresponding author upon request.
